# The Effect of the Dietary Approaches to Stop Hypertension (DASH) Diet on Sleep, Mental Health, and Hormonal Changes: A Randomized Clinical Trial in Women With Type 2 Diabetes

**DOI:** 10.3389/fnut.2022.775543

**Published:** 2022-05-12

**Authors:** Elnaz Daneshzad, Javad Heshmati, Vahid Basirat, Seyed-Ali Keshavarz, Mostafa Qorbani, Bagher Larijani, Nick Bellissimo, Leila Azadbakht

**Affiliations:** ^1^Non-Communicable Diseases Research Center, Alborz University of Medical Sciences, Karaj, Iran; ^2^Songhor Healthcare Center, Kermanshah University of Medical Sciences, Kermanshah, Iran; ^3^Department of Gastroenterology, School of Medicine, Isfahan University of Medical Sciences and Health Services, Isfahan, Iran; ^4^Department of Clinical Nutrition, School of Nutritional Sciences and Dietetics, Tehran University of Medical Sciences, Tehran, Iran; ^5^Department of Epidemiology, Non-Communicable Diseases Research Center, Endocrinology, and Metabolism Population Sciences Institute, Tehran University of Medical Sciences, Tehran, Iran; ^6^Endocrinology and Metabolism Research Center, Endocrinology and Metabolism Clinical Sciences Institute, Tehran University of Medical Sciences, Tehran, Iran; ^7^School of Nutrition, Ryerson University, Toronto, ON, Canada; ^8^Department of Community Nutrition, School of Nutritional Sciences and Dietetics, Tehran University of Medical Sciences, Tehran, Iran; ^9^Diabetes Research Center, Endocrinology and Metabolism Clinical Sciences Institute, Tehran University of Medical Sciences, Tehran, Iran

**Keywords:** dietary approches to stop hypertension, depression, anxiety, sleep, diabetes

## Abstract

**Background:**

Some dietary patterns may improve diabetes complications through scavenging oxidants and anti-inflammatory properties. This study evaluated the effect of the Dietary Approaches to Stop Hypertension (DASH) diet on sleep status, mental health, and hormonal changes among Iranian women with type 2 diabetes.

**Methods:**

This randomized controlled trial (RCT) included 66 diabetic women. Participants were randomly divided into the two different diet groups (the DASH diet and control diet; 33 patients in each group) for 3 months. The Pittsburgh Sleep Quality Index and the Depression, Anxiety, and Stress Scale-21 items were used to assess sleep and mental disorders, respectively. Fasting blood sugar, hemoglobin A1c (HbA1c), advanced glycation end products (AGEs), as well as several sex hormones were evaluated at the beginning and the end of the trial.

**Results:**

Anthropometric indices, HbA1c (control: 8.77 ± 0.82 vs. 8.04 ± 1.03; the DASH diet 8.70 ± 1.05 vs.7.41 ± 1.03), and follicle-stimulating hormone (FSH) (control: 72.16 ± 26.02 vs. 68.12 ± 27.63; the DASH diet: 72.99 ± 25.19 vs. 67.43 ± 27.63) significantly decreased over 12 weeks in both the groups (*P* < .0001). Testosterone, 2-h postprandial glucose (2hPPG), and AGEs significantly decreased over 12 weeks in the DASH diet group. Sleep, depression, and anxiety scores significantly decreased over 12 weeks in the DASH diet group. Night sleep duration significantly increased over 12 weeks in the DASH diet group (*P* < 0.0001).

**Conclusion:**

A 12-week DASH diet significantly decreases testosterone, 2hPPG, AGEs level, as well as sleep, depression, and anxiety scores in women with type 2 diabetes. However, more RCTs are needed to confirm these findings.

## Introduction

Diabetes is a critical non-communicable disease and a severe worldwide public health problem. The WHO has predicted that diabetes is projected to affect 366 million people by 2030 (1). A complex interaction between genetic predisposition, environmental factors, and lifestyle factors such as inactivity and unhealthy diets can contribute to increased risk of diabetes mellitus (2).

Diabetes is associated with various complications such as micro- and macrovascular disorders, anxiety, depression, and sleep disturbances. Psychological disorders are a major cause of disability and lead to poor social outcomes (3). One-third of people with diabetes experience moderate-to-severe levels of depression, anxiety, or both. Diabetes may also act as a trigger for mental health problems, while mental disorders can interfere with optimal diabetes self-management, leading to poorer glycemic control (4). Moreover, several studies have reported that sleep apnea and sleep deprivation could occur in diabetes (5). On the other hand, sleep deprivation is associated with some other problems and chronic diseases such as cardiometabolic disorders, depression, and elevated mortality rates (6–8). Hyperglycemia increases reactive oxygen species (ROS) in diabetic patients, which cause increased oxidative stress (9). Increasing oxidative stress status and imbalance of oxidative and antioxidative status may lead to psychological distress and sleep disorders (10). Increasing the antioxidant capacity of the diet could be a useful approach to control these severe complications in diabetes. Also, healthy dietary patterns, which are rich in vegetables and low in fat, showed a positive association with longer sleep and good sleep quality (11–13).

Some previous studies have shown that higher consumption of refined grains, sugar-sweetened beverages, saturated fatty acids, and processed meats are associated with psychological disorders. Other studies have shown that consumption of legumes, nuts, vegetables, fruits, and unrefined grains seem to be associated with a reduced risk of depression and anxiety (14, 15). The Dietary Approaches to Stop Hypertension (DASH) diet recommends the high intake of low-fat dairy products, whole grain, fruits and vegetables, and sodium restriction. This diet is a low-glycemic index and low-energy-dense dietary pattern, which is high in unsaturated fatty acids, fiber, and antioxidant components (16). The DASH diet contributes as a source of the healthy food groups containing various nutrients such as magnesium, potassium, and phytoestrogens, which have beneficial effects on glycemic control and insulin sensitivity (17). Studies have shown beneficial effects of the DASH diet on levels of blood glucose and blood lipids among patients with type 2 diabetes mellitus (T2DM) (16). A meta-analysis of randomized clinical trials has shown that the DASH diet can reduce fasting insulin concentration in the intervention group among diabetic patients (17). Most available studies on the effectiveness of the DASH diet have been focused on glycemic and cardiovascular risk factors. There is a cross-sectional study that showed health benefits of the DASH diet score with sleep duration and quality (18). This is the first study that aimed to evaluate the effect of the DASH diet (intervention) on sleep status and mental health (primary outcomes) among Iranian patients with T2DM. Previous studies have shown that depression can affect sex hormones concentration in females and estrogen has a positive effect on mood in middle-aged women (19, 20). Another study showed that lower levels of sex hormone-binding globulin (SHBG) are associated with increasing obstructive sleep apnea (21). Due to limited evidence of sexual hormones and health outcomes, we examined the effect of the DASH diet on changes in sex hormones as secondary outcomes. Sleep and mental disorders are more prevalent among women, especially in older and postmenopausal women (22, 23). Sex-related differences in diabetes prevalence and its associated risk factors have been previously assessed (24, 25) and mental and sleep status can be affected during different phases of menopause. Moreover, it seems that decreases in sex hormones such as estrogen and progesterone are associated with depression, anxiety, and sleep apnea during postmenopausal period (26).

## Methods

### Subjects

This parallel randomized clinical trial was conducted among 66 postmenopausal females with T2DM who were referred to a diabetes clinic at Tehran University of Medical Sciences (TUMS), Tehran, Iran. This study was conducted from July 2018 to March 2019. This study was approved by the ethics committee of TUMS (Code: 36923). Also, we registered the trial at the Iranian Registry of Clinical Trials (Registration number: IRCT20180312039055N1, Registration date: 20/05/2018; trial ID: 30485). The sample size was calculated based on the following formula using advanced glycation end products (AGEs) variable: (Z_1−α/2_ + Z_1−β_)^2^ + ($\sigma_{1}^{2}$ + $\sigma_{2}^{2}$)^2^/(μ_2_-μ_1_)^2^ = (1.96 + 0.84)^2^ + (455 + 150)^2^/(520–276)^2^ = 30 (27), as well as SHBG: *n* = (Z1–α/2 + Z1–β)^2^ + (σ12 + σ22)/(μ2–μ1)^2^ = (1.96 + 1.28)^2^ + (18.82 + 21.71)^2^/(28.80–11.66)^2^ = 30 (28). Based on this formula, 30 subjects were needed in each group to have adequate power. However, because of dropout, we continued the sampling process to 33 in each group.

Inclusion criteria were as follows: postmenopausal women with T2DM, willing to participate in the trial, history of taking a stabilized dose of oral antidiabetic medication, and no history of taking supplements within 3 months. The exclusion criteria were any change in medical treatments during the trial and if participants did not return to the clinic for follow-up visits. Also, patients who were receiving medical treatment, including antiobesity, anti-inflammatory, or antipsychotics, insulin therapy, hormone therapy, having other clinical diseases such as type 1 diabetes, liver and kidney damage, asthma, malignancy and cancers, gastrointestinal diseases, food allergy, hormonal disorders, pancreatitis, and thyroid dysfunction, were not included in this study. All the patients declared their participation in the project by providing a written informed consent.

After the screening procedure, we used the permuted block randomization method to allocate subjects into the 2 groups (the DASH or control diet; 33 patients in each group). For matching patients, we used stratified randomization based on body mass index (BMI) (normal, overweight, and obese). Hence, the intervention was a diet therapy and we could not perform a concealing procedure. Although participants were not blind to treatment, the laboratory assessors were blinded to group allocation and study hypothesis.

A questionnaire was used for sociodemographic information. Subjects were required to record their 3-day physical activity (PA) every 2 weeks seven times. Finally, PA levels were expressed by the metabolic equivalent hour per day (MET-h/day) (29).

### Dietary Intervention

The intervention group (33 patients) received the DASH diet and the other 33 patients received a control diet for 3 months. Both the diets included a macronutrient composition of 50–55% carbohydrates, 15–20% protein, and 30% total fat. The DASH diet was rich in whole grains, low-fat dairy products, vegetables, and fruits. It was low in sweets, refined grains, total fat, cholesterol, and saturated fat. The control diet presented as a usual diet with no recommendation for increasing consumption of whole grains, fish, and nuts, as well as no recommendation for fat intakes restriction. The control group consumed less serving of nuts, whole grains, low-fat dairy, fruits, vegetables, and whole grains compared to the intervention group. Table 1 presents serving sizes of the daily food groups and the main differences between the DASH and control diets based on a 2,000-kcal diet. The amount of sodium was no more than 2,400 mg per day. The prescribed calorie was the same for both the groups. Each intervention group received a list of approved foods with a 7-day menu, as well as an exchange list to ensure diversity of food consumption. We checked the compliance of subjects as follows:

- Dietary records. All the participants were asked to provide seven 3-day food records (every 2 weeks during the trial), which consisted of serving sizes of consumed foods with ingredients. Household measures were used to obtain grams of food consumed (30). Nutritionist IV, which is adapted for Iranian foods, was used to perform nutrient analysis (First Databank Division, The Corporation, San Bruno, California, USA).
- Weekly phone calls.
- Serum level of vitamin C during the first and last visits of this study.

**Table 1:** Daily food groups serving sizes for diabetic patients on a 2,000-kcal diet.

**Groups**	**DASH diet**	**Control diet**
Grains	7 (whole grains)	10
Fruits	5	3
Vegetables	5	3
Dairy	3 (low-fat types)	3
Meats	2 (chicken and fishes)	6
Nuts	2	0–0.5
Fat and oils	3	7

*DASH, dietary approaches to stop hypertension*.

We asked patients in both the groups to sustain their physical activity during this study, which was confirmed through weekly phone calls and reviewing the patients seven 3-day physical activity records.

### Assessment of Anthropometric Measures

All the anthropometric measurements were measured at the beginning and end of the trial. Weight was recorded in minimally clothed participants and without footwear using a calibrated digital scale (SECA 803, Germany). Height was measured in a standing position by using an upstretched tape measure, while shoulders were in the normal position. BMI was calculated as weight divided by height squared (kg/m^2^). Waist circumference (WC) was measured at the narrowest part of the waist between the last rib and the iliac crest over light clothing, using an unscratched anthropometric tape to the nearest 0.1 cm.

### Assessment of Biochemical Tests

Blood samples (10 ml of venous blood) were collected at the beginning and end of this study after a 12-h overnight fasting to determine serum levels of fasting blood sugar (FBS), advanced glycation end products (AGEs), testosterone, sex hormone-binding globulin (SHBG), luteinizing hormone (LH), follicle-stimulating hormone (FSH), and vitamin C. Serum samples were separated after centrifuging at 3,000 rpm for 20 min and stored at −80°C. However, FBS was measured on the day of blood collection by enzymatic colorimetric method using glucose oxidase. Hemoglobin A1c (HbA1c) was measured using the immunoturbidimetric assay in whole blood. ELISA kits were used for measuring AGEs (Crystal day, China), testosterone (PadtanGostar Isar, Iran), SHBG (DiaMetra, Italy), LH (Pishtaz Teb, Iran), FSH (Pishtaz Teb, Iran), and vitamin C (Crystal day, China).

### Assessment of Depression, Anxiety, and Stress

The Depression, Anxiety, and Stress Scale-21 items (DASS-21) was used to assess psychological symptoms (31). This self-reported scale has three subscales (contain 7 questions for each subscale), including depression, anxiety, and stress, which were assessed through 21 questions. Each question was scored from 0 to 3 and scores from each subscale were summed. For depression, a total score of zero to 9 is normal, whereas scores above 10 relate to increasing severity of depression (mild to extremely severe). For anxiety, a total score of zero to 7 is normal and scores above 8 relate to increasing severity of anxiety (mild to extremely severe). For stress, a total score of zero to 14 is normal, whereas scores 15 and above relate to increasing severity of stress (mild to extremely severe). Finally, each subject was classified into either normal or abnormal levels of psychological disorders.

### Assessment of Sleep Duration

Participant sleep duration was extracted from sociodemographic information. Moreover, all the participants were asked to complete the validated self-report Pittsburgh Sleep Quality Index (PSQI) (32). The PSQI measures the pattern and quality of sleep over the past month and consists of 9 items that explain sleep latency, duration, and efficiency, using sleep medication, sleep disturbances, and daytime dysfunction. These items differentiate poor to good value with a range of 0 to 3 (0, not in the past month; 1, less than once per week; 2, once or twice per week; and 3, three or more times per week). The PSQI scales by a total score of 0 to 21. A score of 5 and above suggests poor sleep quality.

### Statistical Analysis

All the statistical analyses were performed using the Statistical Package for Social Sciences (version 16.0; SPSS Incorporation, Chicago, Illinois, USA). The normality of data distribution was assessed by using the Kolmogorov–Smirnov test and also histogram curve. Independent sample *t*-test was used for determining mean differences between the groups for continuous variables such as age, weight, and body mass index. The chi-squared test was used to determine the distribution of categorical variables between the intervention and control groups. The between-group analysis was determined using repeated measure analysis of covariance (ANCOVA). Adjustments were conducted for baseline values, age, protein intake, socioeconomic status, weight reduction, triglyceride changes, and blood glucose changes in different models. *P* < .05 was considered as statistically significant.

## Results

**Figure 1:**
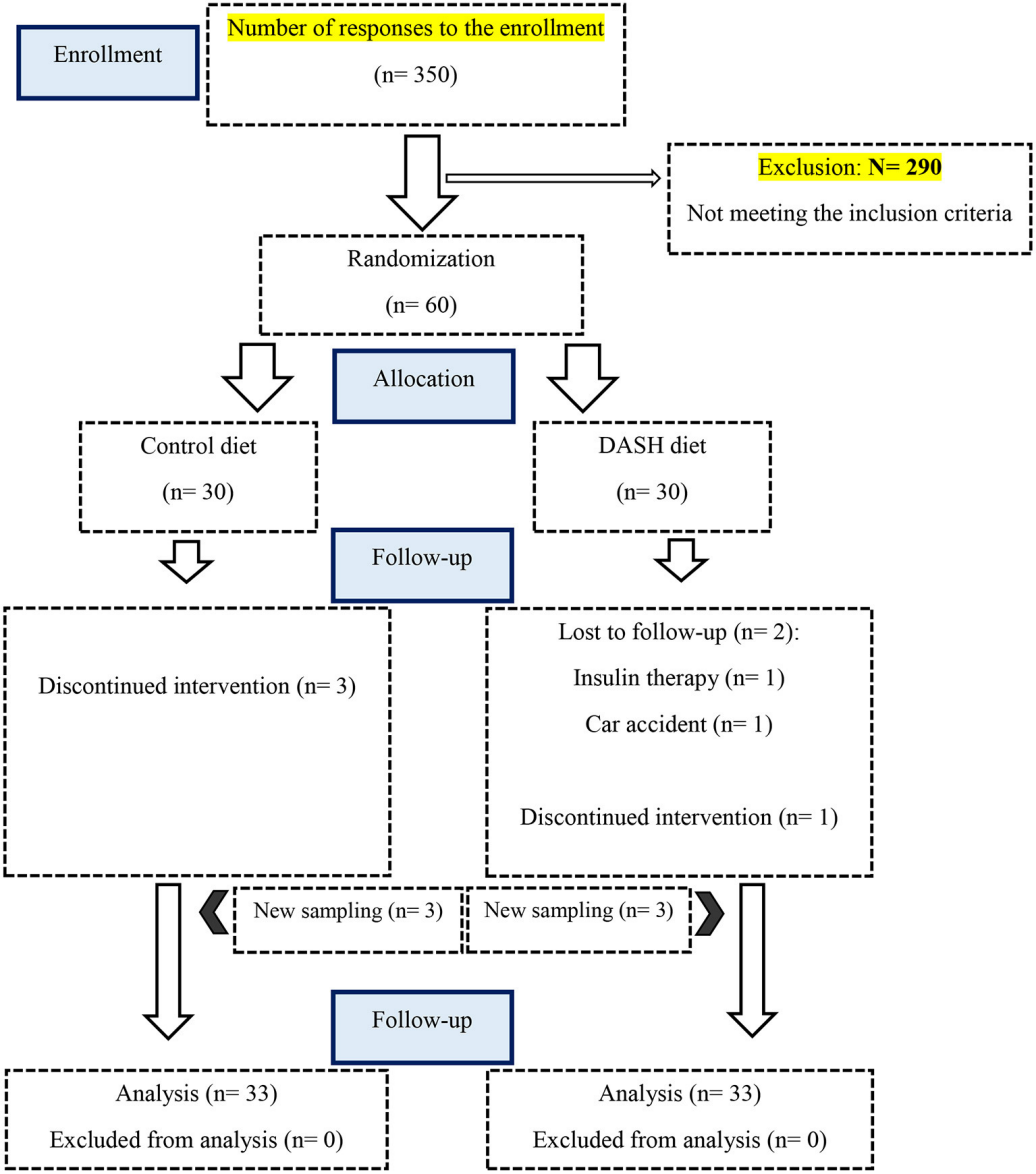
Study flowchart.

Data from 66 participants were analyzed and included in the intention-to-treat analysis (*n* = 66). Therefore, based on recruiting 33 per group rather than 30 per group, the attrition rate was 10%. Figure 1 shows the flow diagram of the trial in this study. Three individuals in the intervention group and 3 individuals in the control group failed to complete the trial. Three subjects in the control group did not desire to complete the trial. In the intervention group, one participant did not desire to finish the trial, 1 participant had a car accident, which led to an inflammatory condition, and 1 participant was excluded due to shifting their medication therapy to insulin therapy. Baseline characteristics in each group are shown in Table 2. The mean age of patients in the DASH diet (57.52 ± 4.99 years) was significantly lower than the control group (60.70 ± 6.33 years) (*P* =0.027). Also, socioeconomic status score was significantly higher among the DASH diet (19.33 ± 3.76) rather than the control group (15.78 ± 4.05) (*P* <0.0001). There were no significant differences in waist circumference (the DASH diet: 98.92 ± 7.92 cm vs. the control group: 101.16 ± 8.96 cm) (*P* = 0.286), BMI (the DASH diet: 29.83 ± 4.25 kg/m^2^ vs. the control group: 29.31 ± 3.67 kg/m^2^) (*P* = 0.594), and physical activity (the DASH diet: 28.91 ± 3.42 MET-h/day vs. the control group: 27.48 ± 2.36 MET-h/day) (*P* = 0.092) among the intervention and control groups at baseline. As previously shown in Table 1, the intervention group was recommended to consume more vegetables, fruits, grains, especially whole grains and nuts, and to consume lower red and processed meat and fats rather than the control group. These recommendations were applied to find the effect of the DASH diet on primary and secondary outcomes and as shown in Table 3, there was significant difference between the consumed food groups by the intervention and control groups. Dietary intakes of participants during the 3-month intervention period are shown in Table 3. Energy, carbohydrate, and total fat intakes were not significantly different among the DASH diet and the control group (*P* ≥.05). The DASH diet group consumed more potassium (the DASH diet: 4272.53 ± 210.39 mg vs. the control group: 2355.98 ± 267.03 mg), fiber (the DASH diet: 26.14 ± 2.66 g vs. the control group: 14.61 ± 3.47 g), and vitamin C (the DASH diet: 261.01 ± 53.37 mg vs. the control group: 90.62 ± 35.44 mg) than the control group per day (*P* < 0.0001).

**Table 2:** Baseline characteristics of women with type 2 diabetes.

**Variables**	**DASH diet (*n* = 33)**	**Control diet (*n* = 33)**	***P*** **-value**
Age (years)	57.52 ± 4.99	60.70 ± 6.33	0.027
BMI (kg/m^2^)	29.83 ± 4.25	29.31 ± 3.67	0.594
WC (cm)	98.92 ± 7.92	101.16 ± 8.96	0.286
WHtR	0.62 ± 0.05	0.64 ± 0.06	0.218
PA (MET-h/d)	28.91 ± 3.42	27.48 ± 2.36	0.092
SES	19.33 ± 3.76	15.78 ± 4.05	< 0.0001
Medication^*^			
All types	11 (33.30)	12 (36.40)	0.796
Just 2 Type	22 (66.7)	21 (63.60)	

*Data are mean ± SD or n (%)*.

*Calculated by independent t-test and chi-square test for quantitative and qualitative variables, respectively*.

* *All types refers to participants who consumed medications for lowering blood glucose, blood pressure and blood lipids, while 2 types refers to participants who just consumed 2 types of mentioned medications*.

*DASH, dietary approaches to stop hypertension; BMI, body mass index; WC, waist circumference; WHtR, waist to height ratio; PA, physical activity; MET, metabolic equivalents; SES: socioeconomic status*.

**Table 3:** Dietary intakes of participants (patients with type 2 diabetes) during 3-month intervention period.

**Daily dietary intake**	**DASH diet (*n* = 33)**	**Control diet (*n* = 33)**	***P*** **-value**
Energy (kcal)	1969.17 ± 72.08	2000.73 ± 101.03	0.149
Protein (% of energy)	17.21 ± 1.13	14.52 ± 1.22	< 0.0001
CHO (% of energy)	55.23 ± 3.73	56.12 ± 3.42	0.315
Total fat (% of energy)	30.91 ± 3.82	30.32 ± 3.41	0.507
Cholesterol (mg)	166.52 ± 27.65	213.46 ± 66.45	0.001
Potassium (mg)	4272.53 ± 210.39	2355.98 ± 267.03	< 0.0001
Sodium (mg)	2391.48 ± 123.02	3821.90 ± 1204.01	< 0.0001
Fiber (g)	26.14 ± 2.66	14.61 ± 3.47	< 0.0001
Vitamin C (mg)	261.01 ± 53.37	90.62 ± 35.44	< 0.0001
Fruits (g)	455.29 ± 57.99	170.72 ± 45.11	< 0.0001
Vegetables (g)	459.54 ± 60.98	197.89 ± 60.36	< 0.0001
Whole grain (g)	127.02 ± 25.05	15.41 ± 15.17	< 0.0001
Nuts (g)	45.62 ± 22.76	9.33 ± 6.47	< 0.0001

*Participants recorded their dietary records for 3 d/wk during weeks 2, 4, 6, 8, 10, and 12 of the trial*.

*Data are mean ± SD. All intakes in grams have been adjusted for total caloric intake. Differences among groups were analyzed using independent t-test*.

*CHO, carbohydrate; DASH, dietary approaches to stop hypertension*.

Table 4 indicates the anthropometric indices and biochemical tests at baseline and after 12 weeks of intervention in postmenopausal diabetic patients. Baseline values for weight (the DASH diet: 73.90 ± 11.29 kg vs. the control group: 72.03 ± 10.11 kg), WC (the DASH diet: 98.92 ± 7.92 cm vs. the control group: 101.16 ± 8.96 cm), BMI (the DASH diet: 29.77 ± 4.11 kg/m^2^ vs. the control group: 29.28 ± 3.61 kg/m^2^), LH (DASH diet: 23.69 ± 13.23 IU/l vs. the control group: 21.06 ± 12.61 IU/l), FSH (the DASH diet: 72.99 ± 25.19 IU/l vs. the control group: 72.16 ± 26.02 IU/l), SHBG (the DASH diet: 22.22 ± 10.01 nmol/l vs. the control group: 24.96 ± 12.91 nmol/l), testosterone (the DASH diet: 0.83 ± 0.41 nmol/l vs. the control group: 0.79 ± 0.37 nmol/l), FBS (the DASH diet: 153.60 ± 40.52 mg/dl vs. the control group: 155.93 ± 44.19 mg/dl), HbA1c (the DASH diet: 8.70 ± 1.05% vs. the control group: 8.77 ± 0.82%), 2hPPG (the DASH diet: 198.87 ± 60.59 mg/dl vs. the control group: 195.42 ± 60.50 mg/dl), AGEs (the DASH diet: 351.58 ± 215.11 ng/l vs. the control group: 298.34 ± 222.96 ng/l), and serum vitamin C (the DASH diet: 30.58 ± 6.96 ng/l vs. the control group: 29.52 ± 8.34 ng/l) were not significantly different between the intervention and control groups (*P* > 0.05). At the end of the trial, there was a significant difference among the groups for all the abovementioned factors, except for WC (*P* = 0.591), LH (*P* = 0.760), and FSH (*P* = 0.427). Weight, WC, BMI, HbA1c, and FSH significantly decreased over 12 weeks in both the groups (*P* < 0.0001). Testosterone, 2hPPG, and AGEs significantly decreased over 12 weeks in the DASH diet group (*P* < 0.0001). Table 5 depicts sleep, stress, anxiety, and depression scores at baseline and after 12 weeks of intervention in diabetic women.

**Table 4:** Anthropometric indices and biochemical tests at baseline and after 12 weeks of intervention in postmenopausal diabetic patients.

**Variable**	**DASH diet (*n* = 33)**	**Control diet (*n* = 33)**	***P*** **-value**	**ES**
Weight (kg)				
Baseline	73.90 ± 11.29	72.03 ± 10.11	0.474 ^a^	
End of trial	69.23 ± 9.47	70.02 ± 9.47	0.006 ^b^	0.144
BMI (kg/m^2^)				
Baseline	29.77 ± 4.11	29.28 ± 3.61	0.606 ^a^	
End of trial	28.30 ± 4.53	28.50 ± 4.53	0.001 ^b^	0.001
WC (cm)				
Baseline	98.92 ± 7.92	101.16 ± 8.96	0.286 ^a^	
End of trial	94.90 ± 10.34	96.94 ± 10.34	0.591 ^b^	0.073
FBS (mg/dl)				
Baseline	153.60 ± 40.52	155.93 ± 44.19	0.824 ^a^	
End of trial	128.05 ± 45.43	155.29 ± 45.43	0.028 ^c^	0.004
2-hPPG (mg/dl)				
Baseline	198.87 ± 60.59	195.42 ± 60.50	0.817 ^a^	
End of trial	158.37 ± 73.01	200.15 ± 73.01	0.040 ^c^	0.073
HbA1c (%)				
Baseline	8.70 ± 1.05	8.77 ± 0.82	0.751^a^	
End of trial	7.41 ± 1.03	8.04 ± 1.03	0.029 ^c^	0.136
LH (IU/L)				
Baseline	23.69 ± 13.23	21.06 ± 12.61	0.412^a^	
End of trial	26.08 ± 15.10	24.07 ± 15.10	0.760 ^d^	0.023
FSH (IU/L)				
Baseline	72.99 ± 25.19	72.16 ± 26.02	0.896 ^a^	
End of trial	67.43 ± 27.63	68.12 ± 27.63	0.427 ^d^	0.017
SHBG (nmol/l)				
Baseline	22.22 ± 10.01	24.96 ± 21.91	0.597 ^a^	
End of trial	30.64 ± 27.11	18.55 ± 27.11	0.016 ^d^	0.124
Testosterone (nmol/l)				
Baseline	0.83 ± 0.41	0.79 ± 0.37	0.667 ^a^	
End of trial	0.63 ± 0.34	0.87 ± 0.34	0.040 ^d^	0.083
AGEs (ng/L)				
Baseline	351.58 ± 215.11	298.34 ± 222.96	0.327 ^a^	
End of trial	234.67 ± 241.21	367.88 ± 241.21	0.001 ^e^	0.261
Vitamin C (ng/L)				
Baseline	30.58 ± 6.96	29.52 ± 8.34	0.579 ^a^	
End of trial	33.71 ± 7.01	29.22 ± 7.01	< 0.0001	0.187

*Data are mean ± SD*.

*ES, effect size; WC, waist circumference; FBS, fasting blood sugar; 2-hPPG, 2 h post prandial glucose; DASH, dietary approaches to stop hypertension; LH, luteinizing hormone, FSH, follicular stimulating hormone; SHBG, sex hormone binding globulin; AGEs, advanced glycated end products*.

a *Baseline differences among two groups presented using independent T-test*.

b *P-value values show a comparison of final values among two groups using two way repeated-measure analysis of variance (Adjustment has run for age, protein intake, baseline values and socio-economic status)*.

c *P-value values show a comparison of final values among two groups using two way repeated-measure analysis of variance (Adjustment has run for age, protein intake, weight reduction, baseline values and socio-economic status)*.

d *P-value values show a comparison of final values among two groups using two way repeated-measure analysis of variance (Adjustment has run for age, protein intake, weight reduction, triglyceride changes, baseline values and socio-economic status)*.

e *P-value values show a comparison of final values among two groups using two way repeated-measure analysis of variance (Adjustment has run for age, protein intake, weight reduction, triglyceride changes, blood glucose changes, baseline values and socio-economic status)*.

**Table 5:** Sleep, stress, anxiety, and depression scores at baseline and after 12 weeks of intervention in postmenopausal diabetic patients.

**Variable**	**DASH diet (*n* = 33)**	**Control diet (*n* = 33)**	***P*** **-value**	**ES**
Sleep score				
Baseline	16.27 ± 5.06	15.60 ± 5.26	0.536 ^a^	
End of trial	8.66 ± 5.80	15.91 ± 5.80	< 0.0001 ^b^	0.257
Night sleep duration (hour)				
Baseline	6.61 ± 1.09	6.27 ± 1.39	0.272 ^a^	
End of trial	7.53 ± 1.20	6.18 ± 1.20	0.009 ^b^	0.107
Depression score				
Baseline	6.66 ± 3.39	5.40 ± 3.42	0.135 ^a^	
End of trial	3.19 ± 3.44	5.97 ± 3.44	< 0.0001 ^b^	0.234
Anxiety score				
Baseline	6.69 ± 3.04	5.57 ± 3.10	0.143 ^a^	
End of trial	3.96 ± 3.50	5.24 ± 3.50	0.026 ^b^	0.135
Stress score				
Baseline	10.12 ± 4.05	10.00 ± 3.33	0.895 ^a^	
End of trial	4.61 ± 3.27	8.05 ± 3.27	0.005 ^b^	0.167

*Data are mean ± SD*.

*DASH, dietary approaches to stop hypertension; ES: effect size*.

*Baseline differences among groups presented using independent T-test*.

*Two way repeated-measure ANOVA was used to indicate end-of-trial differences among groups*.

a *P-values show a comparison of baseline values among two groups using independent t-test*.

b *P-value values show a comparison of final values among two groups using analysis of variance (Adjustment has run for age, protein intake, weight reduction, baseline values and socio-economic status)*.

Depression, anxiety, stress, and sleep scores, and night sleep duration were not significantly different between the intervention and control groups (*P* > 0.05) at the baseline. However, at the end of the trial, there were significant differences between the groups for all of the foregoing factors. The sleep score was lower in the DASH diet group in comparison to control diet (the DASH diet: 8.66 ± 5.80 vs. the control group: 15.91 ± 5.80). Stress score significantly decreased over 12 weeks in both the groups. Moreover, this reduction in the DASH diet group was significant in comparison to the control group (the DASH diet: 4.61 ± 3.27 vs. the control group: 8.05 ± 3.27). Sleep, depression, and anxiety scores significantly decreased over 12 weeks in the DASH diet group. Night sleep duration significantly increased over 12 weeks in the DASH diet group (*P* < 0.0001).

## Discussion

In this study, we examined the effect of the DASH diet on sleep and psychological status in postmenopausal women with type 2 diabetes. The results suggest that the DASH diet had a beneficial effect on depression, anxiety, and stress, as well as sleep status in type 2 diabetic patients. In a cross-sectional study, Valipour et al. showed that moderate adherence to the DASH diet was negatively associated with depression (33). In this recent cross-sectional study in women with diabetes, patients who adhered to the animal-based dietary patterns were more likely to be poor sleepers or had components of depression, stress, and anxiety (34).

The high antioxidant capacity of the DASH diet leads to reduced oxidative stress, which is related to decreased insulin resistance (35). Moreover, the DASH diet can improve insulin sensitivity independent of weight loss (36). Oxidative stress and inflammation play a role in initiating psychological disorders via altering brain functions (37). The high antioxidant capacity of the DASH diet through consumption of the food groups such as legumes, nuts, vegetables, and fruits can reduce inflammation and oxidative stress and improve brain functioning by affecting synaptic plasticity (38). Healthful-rich plant foods were associated with improved psychological disorders among Iranian women (39). Beezhold et al. showed that a reduction of animal foods and higher consumption of plant-based foods may have beneficial effects on mental health (40). A clinical trial on 75 Iranian women revealed that consumption of high-fiber diets, especially high in whole grains, can improve levels of inflammatory factors and prevent subsequent health outcomes (41).

Vegetables consumed in the DASH diet are good sources of folate and magnesium. These micronutrients play a role in the synthesis of several neurotransmitters, including dopamine, serotonin, and norepinephrine, and may contribute to brain reactions, as well as anti-inflammatory effects (42, 43). Moreover, vitamin D as one of the important ingredients in the DASH diet can improve mood status. A randomized controlled trial of women with T2DM, anxiety, and vitamin D deficiency revealed that vitamin D supplementation for 16 weeks improved mood status (44).

Nuts and beans are the other important foods in the DASH diet, which have beneficial effects through several mechanisms such as ameliorating insulin resistance and improving postmenopausal symptoms. A controlled-feeding study of 50 patients with T2DM showed that daily consumption of cashews for 8 weeks reduced serum insulin (45). In addition, soybeans can promote the expression of estrogen-sensitive genes by binding to estrogen receptors and can reduce hot flashes in postmenopausal women (46). Also, it is well established that sex steroid hormones impact mental health status and mood through neuroprotective and neurodevelopmental processes (47). A cross-sectional study in China found that nut consumption was inversely associated with depression (48). In a large population study of Iranian adults, legumes and nuts consumption were inversely associated with anxiety in adult men. However, there was no significant association between nuts and legumes and anxiety in women. Furthermore, the depressive symptoms were assessed by hospital anxiety and depression scales, not by the DASS-21 (49). Legumes and nuts are high in magnesium content, which is a key nutrient in many brain reactions and metabolism of neurotransmitters. Moreover, they contain fiber, omega-3 fatty acids, folic acid, and other B vitamins, which may have beneficial effects on mood and mental health status (42, 50).

On the other hand, the DASH diet had beneficial effects on anthropometric indices in this study. In line with our results, Fatahi et al. showed that weight-loss diets rich in whole grains, fruits, and vegetables reduced body weight and waist circumference (51). A cross-sectional study in 3,004 adult women showed that anxiety and depression may be related to weight gain (52). However, it should be noted that even after controlling for weight loss, the DASH diet significantly reduced depressive symptoms and sleep status scores.

To the best of our knowledge, there is no study examining the relationship between the adherence to the DASH diet and AGEs. AGEs can be obtained through dietary intakes or can be produced by various reactions such as oxidation of amino acids, lipids, and sugars. AGEs can be generated by oxidative stress even without hyperglycemia. AGEs predispose individuals to inflammation and oxidative stress, which are related to insulin resistance and diabetes and many other chronic diseases such as neurodegenerative diseases, sleep apnea, cardiovascular diseases, and diabetes (53). In this study, the levels of serum AGEs in the patients who adhered to the DASH diet were significantly lower compared to the control group. It was hypothesized that antioxidant capacity of the DASH diet may improve the serum AGEs level. A 3-month calorie-restricted Mediterranean diet in 47 women with overweight and obesity showed a significant reduction in AGEs and insulin resistance (54). Increased intake of antioxidant foods, including vegetables, fruits, nuts, and whole grains and lower intake of red and processed meats, pastries, and sweetened beverages, led to lower intake of AGEs in the Mediterranean diet and may apply to the DASH diet (54).

This study was the first comprehensive study to examining the effect of the DASH diet on several diabetes-related outcomes such as depression, anxiety, stress, sleep status, and the levels of selected sex hormones. However, there are several limitations that should be noted. The reverse causation between diabetes and psychological disorders is unknown. The results are not generalizable to other populations of different ages and gender. Moreover, we did not measure dietary, urinary, or fecal AGEs. Therefore, future studies examining the effect of the DASH diet or other healthy dietary patterns should include a comprehensive assessment of AGEs.

## Conclusion

In conclusion, the DASH diet improved the biochemical markers in the intervention group and had beneficial effects on depression, anxiety, and stress scores, as well as sleep status. Further studies are suggested to confirm our results.

## Data Availability Statement

The original contributions presented in the study are included in the article/supplementary material, further inquiries can be directed to the corresponding author.

## Ethics Statement

The studies involving human participants were reviewed and approved by Tehran University of Medical Sciences. The patients/participants provided their written informed consent to participate in this study.

## Author Contributions

ED designed and LA supervised the study. ED conducted the study. ED and MQ performed the statistical analyses. ED, JH, and VB prepared a first draft of the manuscript. LA, S-AK, and BL finalized the manuscript. NB edited and revised the manuscript. All authors contributed to the article and approved the submitted version.

## Funding

This study is supported by Tehran University of Medical Sciences (grant number: 36923).

## Conflict of Interest

The authors declare that the research was conducted in the absence of any commercial or financial relationships that could be construed as a potential conflict of interest.

## Publisher's Note

All claims expressed in this article are solely those of the authors and do not necessarily represent those of their affiliated organizations, or those of the publisher, the editors and the reviewers. Any product that may be evaluated in this article, or claim that may be made by its manufacturer, is not guaranteed or endorsed by the publisher.
